# Central nervous system effects of 5-HT_7_ receptors: a potential target for neurodegenerative diseases

**DOI:** 10.1186/s10020-022-00497-2

**Published:** 2022-06-20

**Authors:** Alejandro Quintero-Villegas, Sergio Iván Valdés-Ferrer

**Affiliations:** 1 Department of Neurology, Instituto Nacional de Ciencias Médicas y Nutrición Salvador Zubirán, Mexico City, Mexico; 2 Department of Infectious Diseases, Instituto Nacional de Ciencias Médicas y Nutrición Salvador Zubirán, Mexico City, Mexico; 3 Institute of Bioelectronic Medicine, Feinstein Institutes for Medical Research, Manhasset, NY USA

**Keywords:** 5-HT_7_, Alzheimer disease, Parkinson disease, Dementia, Neurodegeneration, Neuroprotection

## Abstract

5-HT_7_ receptors (5-HT_7_R) are the most recently identified among the family of serotonin receptors. Their role in health and disease, particularly as mediators of, and druggable targets for, neurodegenerative diseases, is incompletely understood. Unlike other serotonin receptors, for which abundant preclinical and clinical data evaluating their effect on neurodegenerative conditions exist, the available information on the role of the 5-HT_7_R receptor is limited. In this review, we describe the signaling pathways and cellular mechanisms implicated in the activation of the 5-HT_7_R; also, we analyze different mechanisms of neurodegeneration and the potential therapeutic implications of pharmacological interventions for 5-HT_7_R signaling.

## Introduction

5-hydroxytryptamine (serotonin) 7 receptors (5-HT_7_R) are members of the family of 5-HT receptors identified in 1993, but their functional and pathological implications are incompletely understood (Bard et al. 1993; Lovenberg et al. 1993; Ruat et al. 1993). Like other serotonin receptors, their activation is mediated by G-protein-coupled receptor (GPCR) signaling pathways. 5-HT_7_R is broadly expressed in the central nervous system (CNS), gastrointestinal tract, and other organs, where they potentially regulate different physiological functions including the sleep–wake cycle, body temperature, nociception, and gastrointestinal motility, to name a few (Sanger 2008; Ciranna and Catania 2014; Chang-Chien et al. 2015). The 5-HT_7_R gene is located in the chromosome 10 (q21-124), which contains 3 introns in the coding region, (Gellynck et al. 2013) moreover, 5-HT_7_R is expressed in four different isoforms, being 5-HT_7_R_(A)_, 5-HT_7_R_(B)_ and 5-HT_7_R_(D)_ the ones isolated in humans, and 5-HT_7_R_(A)_, 5-HT_7_R_(B)_ and 5-HT_7_R_(C)_ in rats, with no apparent functional distinction between each isoform (Heidmann et al. 1997). Of interest, 5-HT_7_R presents high homology between species (90%) but little homology with other 5-HT receptors (as low as 50%) (Hannon and Hoyer 2008).

Experimental data suggest that 5-HT_7_R may be an amenable therapeutic target in neurodegenerative disorders. However, no clinical studies have evaluated the role of 5-HT_7_R in neurodegenerative processes, although targeting other serotonin receptors (with drugs such as selective serotonin reuptake inhibitors) has shown little clinical benefit in neurodegenerative conditions (Hüll et al. 2006; Lalut et al. 2017). Here, we aimed to review the potential role of 5-HT_7_R in neurodegeneration and their potential therapeutic implications, based on different in vivo and in vitro pre-clinical studies (Table 1).

**Table 1:** Common agonists and antagonists of 5-HT_7_R used in preclinical studies

Name	Action mechanism	Administration route (dose)	References
AS-19	Selective full agonist	s.c (5 mg/kg), i.t. (5 µL at 100 µM), i.p. (10 mg/kg)	McDaid et al. (2020), Fields et al. (2015), Albayrak et al. (2013)
LP-12	Selective full agonist	i.t. (10µL at 0.02–0.2 nM), cultures (300 nM)	Godínez-Chaparro et al. (2012), Samarajeewa et al. (2014)
LP-44	Selective full agonist	i.p. (1,5 and 10 mg/kg)	Demirkaya et al. (2016)
LP-211	Selective full agonist	i.p. (1,5 and 10 mg/kg), i.p. (0.25 mg/kg), i.p. (0.003–0.3 mg/kg),i.c.v. (0.2 µL at 2–6 mM)	Demirkaya et al. (2016), Liu et al. (2021), Norouzi-Javidan et al. (2016), (Monti et al. 2014)
Methiothepin maleate	Non-specific 5-HT_1/6/7_R agonist	Culture (10 µM)	Soga et al. (2007)
8-OH-DPAT	Non-specific 5-HT_1A/7_R agonist	i.p. (02–0.4 mg/kg and 1.0 mg/kg)	Cassaday and Thur (2019), Odland et al. (2019)
SB-269970	Competitive selective antagonist, quasi-full inverse agonist	i.p. (10 mg/kg)	Perez-García and Meneses (2005), Liu et al. (2021)
SB-258741	Competitive selective antagonist, partial inverse agonist	s.c. (2.3 mg/kg and 3.5 mg/kg)	Pouzet (2002)
SB-258719	Competitive selective antagonist	i.p. (5 mg/kg)	Brenchat et al. (2011)
HBK-15	Competitive non-selective 5-HT_1A/3/7_R antagonist	i.p. (1.25 mg/kg) i.v. (1.25 mg/kg)	Pytka et al. (2018)
Lurasidone	Competitive non-selective 5-HT_2A/7_R antagonist	Microdialsis (3 mg/kg/d)	Okada et al. (2021)

## Methods

We performed a comprehensive search of the PubMed database English language literature to identify all original research and review articles regarding 5-HT_7_R localization, signaling pathways, effectors, its role in health in the central nervous system, and the pathology of selected neurodegenerative diseases. For that purpose, we included the following Medical Subject Headings (MeSH) and main keywords for searches: 5-HT_7_, LP-211, LP-44, LP-21, AS-19, SB-269970, SB-656104-A, 5-HT_7_R mechanism of action, 5-HT_7_R signaling pathway, 5-HT_7_R effect, 5-HT_7_R distribution, 5-HT_7_R neuroprotection, excitotoxicity, oxidative stress, apoptosis, necrosis, unfolded protein response, endoplasmic reticle stress, amyloid β, tau tangles, tau oligomers, α-synuclein, inflammation, Alzheimer’s, Parkinson’s, Huntington’s, Frontotemporal dementia, dementia, and neurodegeneration. We also reviewed the articles cited in the reference lists of the articles identified during the search. The authors independently reviewed the selected articles. The search included articles available from 1993 (when the receptor was originally cloned) to March 2022.

### Distribution in the CNS

5-HT_7_R is broadly expressed in different cell types in the CNS, including neurons, astrocytes, and microglia (Shimizu et al. 1998; Mahé et al. 2005; Guseva et al. 2014). CNS expression of 5-HT_7_R is not homogeneous; the highest expression occurs in the hippocampus, amygdala, and hypothalamus (Thomas and Hagan 2004), and comparatively lower density occurs in the dorsal raphe, caudate nucleus, putamen, and substantia nigra (Martín-Cora and Pazos 2004). In the amygdala, 5-HT_7_R are present primordially in GABAergic interneurons; in other structures, their expression in specific neurons is not clear, hence of critical importance for therapeutic purposes (Kusek et al. 2021). Of biological and clinical relevance, the regional expression of 5-HT_7_R is evolutionarily conserved in mammals (Martín-Cora and Pazos 2004). 5-HT_7_R are also expressed in the digestive tract, aorta, and other tissues, exerting immunomodulatory effects as well as other organ-specific effects (Quintero-Villegas and Valdés-Ferrer 2019).

### Cellular mechanisms and signaling pathways

Like other GPCR, 5-HT_7_R are found in the cell membrane, where they form homodimers and homoligomers, with no known relevant differences between their biological functions (Smith et al. 2011; Guseva et al. 2014). In addition, 5-HT_7_R can form heterodimers and heterooligomers with 5-HT_1A_ receptors (5-HT_1A_R), which in turn lead to diminished activity and increased internalization of 5-HT_1A_ receptors without a discernible effect on 5-HT_7_R signaling or activity (Renner et al. 2005; Prasad et al. 2019). This is of biological relevance, as activation of 5-HT_1A_R results in activation of G_αi_ and reducing levels of cyclic adenosine monophosphate (cAMP) and mitogen-activated protein kinase (MAPK), also known as an extracellular signal-regulated kinase (ERK), hence antagonizing the effects of the 5-HT_7_R-Gαs signaling cascade (Zhou et al. 2019).

**Fig. 1:**
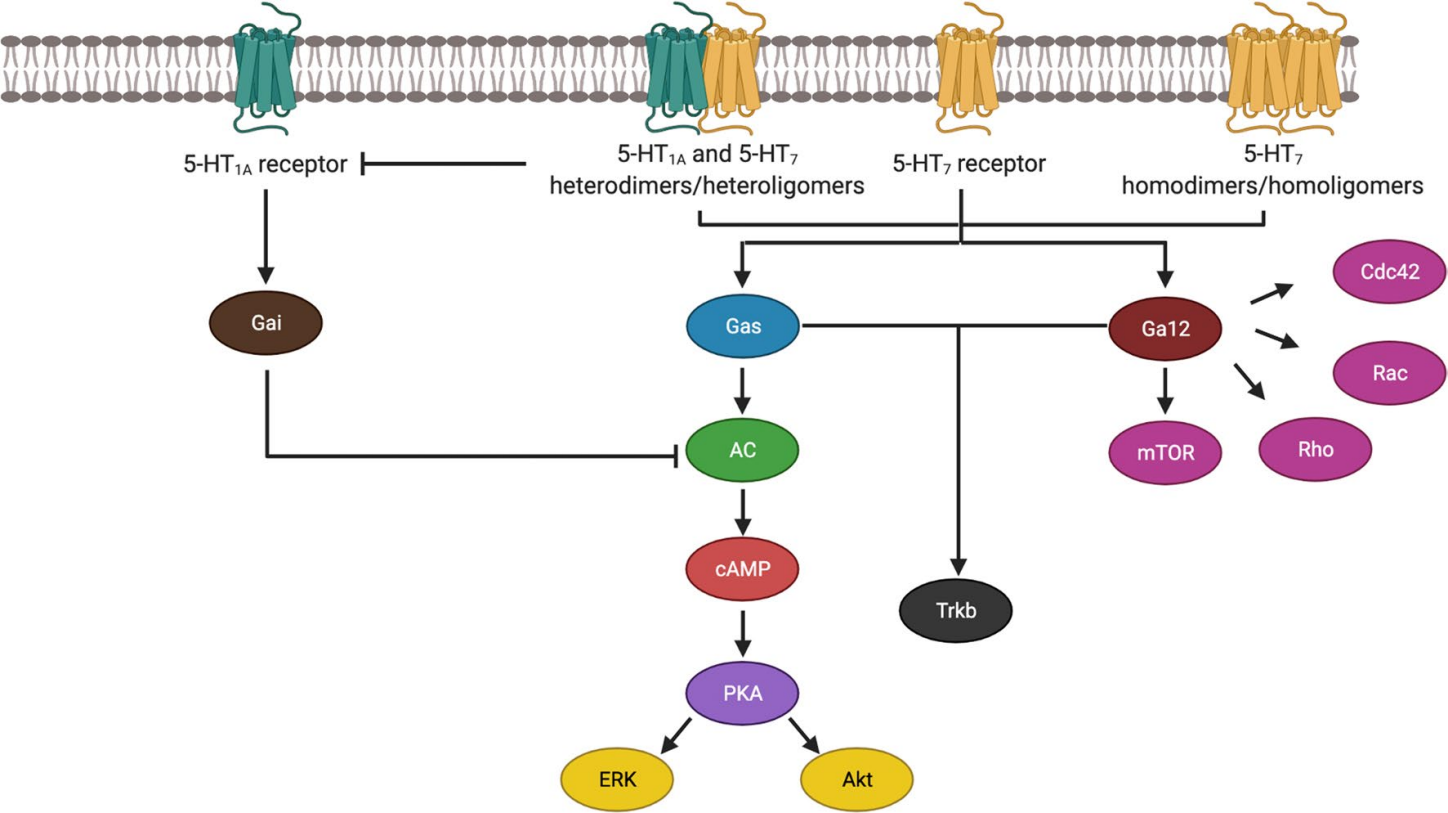
5-HT_7_ and 5-HT_1A_ receptor signaling pathways and oligo/heterodimer formation. 5-HT_7_ receptor monomers (in yellow) can form homodimers or homoligomers, with the same signaling pathways and cellular effects. 5-HT_7_ can also form heterodimers or heteroligomers with 5-HT_1A_ (in teal), resulting in the inhibition of the 5-HT_1A_ signaling pathway, with no net effect downstream of 5-HT_7_. When activated, 5-HT_7_ activates Gas (canonical pathway) with a subsequent signaling cascade that results in the activation of ERK (also known as MAPK) and Akt; in contrast, the activation of Ga12 activates mTOR and different Rho family small GTPases. As illustrated, the phosphorylation of Trkb is mediated by both G proteins. *AC* adenylate cyclase, *cAMP* cyclic adenosine monophosphate, *Cdc42* cell division control protein 42 homolog, *ERK* extracellular signal-regulated kinases, *MAPK* mitogen-activated protein kinases, *mTOR* mammalian target of rapamycin, *Trkb* Tropomyosin receptor kinase B

The activation of 5-HT_7_R leads to the initiation of two possible signaling pathways: the *canonical* one, described when the receptor was originally cloned (Lovenberg et al. 1983) acts via Gαs (Fig. 1). The activation of this pathway, like in other GPCRs results in the phosphorylation of different adenylyl cyclases (AC) (Baker et al. 1998). This leads to cAMP production, activation of protein kinase A, and, finally, phosphorylation of different proteins, like ERK 1 and ERK 2, Akt, and tropomyosin-related kinase B (Trkb) (Errico et al. 2001; Johnson-Farley et al. 2005; Samarajeewa et al. 2014).

The *Non-canonical* signaling pathway of 5-HT_7_R acts via Gα12 (Guseva et al. 2014). This leads to the activation of Rho, Rac, and cell division control protein 42 (Cdc42) all part of the Rho family of small GTPases, which in neurons promote dendrite sprouting, formation of filopodia, and synaptogenesis (Hart, et al. xxxx; Kobe et al. 2012a; Speranza et al. 2013; Speranza et al. 2015; Marin and Dityatev 2017). Of relevance, Trkb expression (a brain-derived neurotrophic factor [BDNF] receptor) appears to be enhanced by both Gαs and Gα12 (Fig. 1) (Samarajeewa et al. 2014). These signaling pathways may be of therapeutic relevance for neurodegenerative diseases, although few studies have so far evaluated these effects (Hashemi-Firouzi et al. 2017; Costa et al. 2018; Quintero-Villegas et al. 2019).

### Biopathology of neurodegeneration

**Fig. 2:**
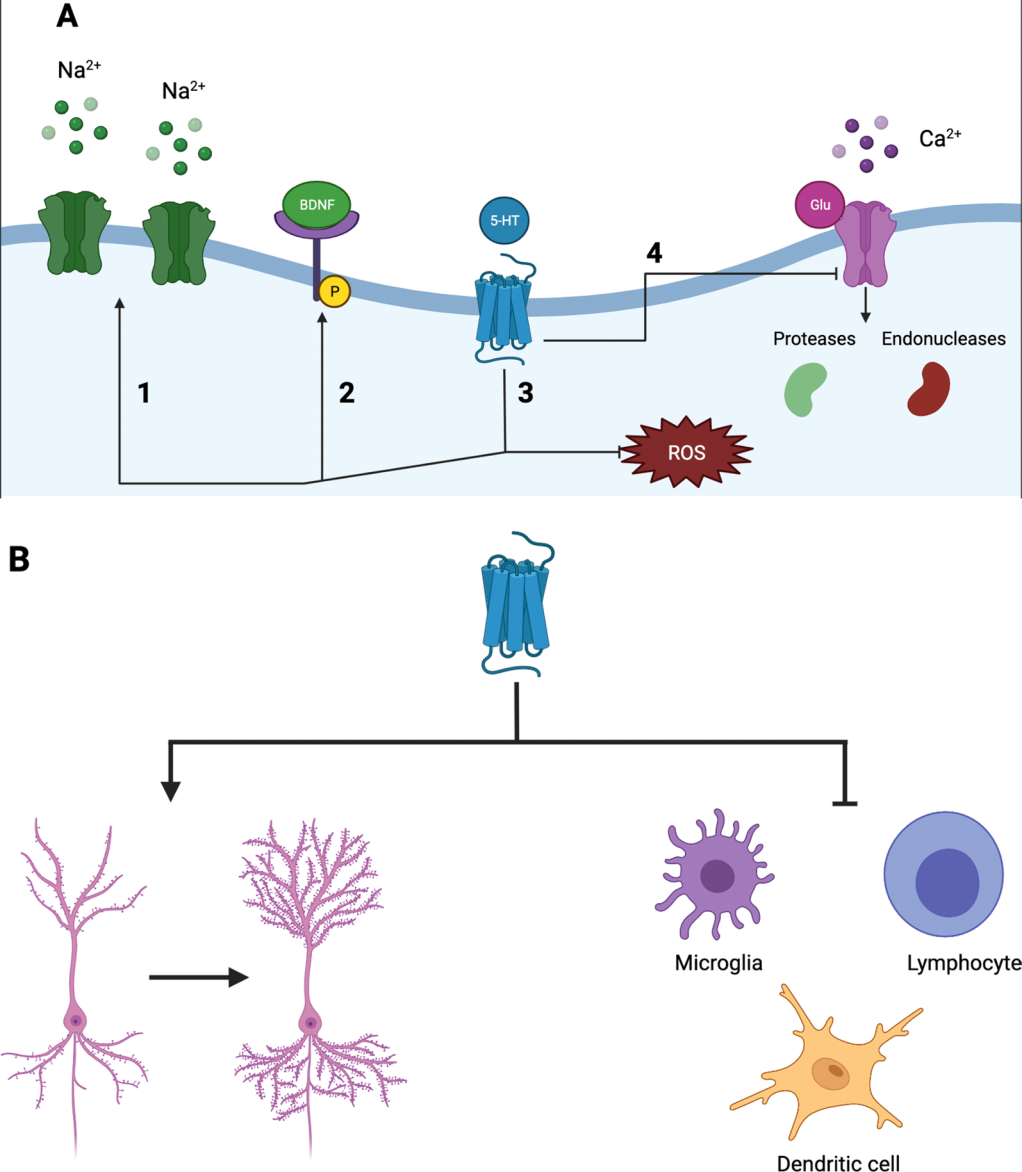
Cellular and molecular effects of 5-HT_7_ receptors. Molecular effects of 5-HT_7_ activation. **A** When activated, 5-HT7 receptors modulate ion transmission through enhancing LTP and LTD (1); these receptors also increase the number of neurotrophins (especially BDNF) and the affinity of its receptor Trkb (2); through ERK and Akt, 5-HT_7_ decreases neuronal damage mediated by ROS (3); and reduces the excitotoxicity burden mediated by glutamate-NMDA-calcium. Cellular effects of 5-HT_7_ activation. **B** When stimulated by serotonin, 5-HT_7_ enhances dendritic sprouting and synaptogenesis, while regulating (often towards suppression) immune cells. *LTD* long-term depression, *LTP* long-term potentiation, *NMDA* (*N*-methyl-d-aspartate receptor, *ROS* reactive oxygen species, *Trkb* tropomyosin receptor kinase B

The biological mechanisms leading to neurodegeneration include many neuronal and glial molecular pathways that result in neuronal damage. Below, we briefly summarize the most common mechanisms involved in neurodegeneration and how they might associate with the known 5-HT_7_R effects (Fig. 2; Table 2).

**Table 2:** Mechanisms of neuronal damage, and possible beneficial effects of 5-HT_7_ receptors agonism

Neurodegeneration mechanism	5-HT_7_ possible role	References
Excitotoxicity	Activation of MAPK/ERK and PI3/Akt/GSK3b protects against glutamate-induced damage Decreased expression of NR2B and NR1 subunits of NMDA glutamate receptors Increased expression of superoxide dismutase and glutathione	Jiang et al. (2000); Pi et al. (2004); Li et al. (2005) Vasefi et al. (2013a) Yuksel et al. (2019)
Oxidative stress	No study evaluated effect in the CNS In a sepsis-induced lung injury, 5-HT_7_ receptor agonism decreased ROS burden 5-HT_7_ antagonism decreased oxidative burden in bleomycin-induced pulmonary fibrosis 5-HT_7_ activation enhances microsome stability towards oxidative metabolism (Lacivita et al., 2016a) ERK and Akt protect PC12 cells from oxidative damage	Cadirci et al. (2013) Tawfik and Makary (2017) Lacivita et al. (2016a) Ong et al. (2016)
Apoptosis	5-HT_7_ receptor agonism reduces apoptosis in the streptozotocin-induced AD model	Hashemi-Firouzi et al. (2017)
Long term depression/ potentiation impairment	5-HT_7_ KO mice display LTP impairment 5-HT_7_ agonism reduces mGluR-dependent LTD	Roberts et al. (2004) Costa et al. (2012)
Synaptic impairment	5-HT_7_ agonism increases dendritic density and synaptogenesis in the cortical and striatal forebrain 5-HT_7_ agonism induces dendritic sprouting and neurite enlargement	Speranza et al. (2013, 2015, 2017) Kvachnina et al. (2005); Canese et al. (2015)
Neurotrophin depletion	5-HT_7_ agonism increases PDGF-β 5-HT_7_ agonism increases the expression and affinity of trk-B	Vasefi et al. (2013b) Samarajeewa et al. (2014)

#### Amyloid β-mediated neurodegeneration

Amyloid β (Aβ) is a key mediator of neurodegeneration in Alzheimer’s disease (AD), inducing damage through multiple pathways (Querfurth and Laferla 2018), some of which overlap with other mechanisms discussed below. Besides AD, Aβ may also play a role in diseases such as frontotemporal dementia (FTD), cerebral amyloid angiopathy, and cerebral amyloidosis (Miller-Thomas et al. 2016). Aβ is a critical source of reactive oxygen species (ROS) and reactive nitrogen species in AD, causing neuronal lipoperoxidation in neurons and thus, neurodegeneration (Bernal-Mondragón et al. 2013). Also, Aβ helps the formation of voltage-independent, cation channels in the lipid membranes, which could lead to excitotoxicity-mediated neurodegeneration (Arispe et al. 1993).

Chronically Aβ-stimulated microglia releases multiple pro-inflammatory cytokines, such as interleukin (IL)-1, IL-6, and tumor necrosis factor (TNF)-α, which induces pathological changes in the CNS (Heppner et al. 2015). Aβ-stimulated microglia increases neuronal damage and further accumulation of Aβ, an effect mediated by receptors for advanced glycation end products (Fang et al. 2010). Although not directly associated with neuronal death, Aβ impairs synaptic function and synaptogenesis and dysregulates neurotransmitter levels in the synaptic cleft, contributing to the symptoms in AD (Querfurth and Laferla 2018; Cai 2019; Ding et al. 2019).

5-HT_7_R agonism with LP-211 (a highly selective agonist) reverts neuronal damage and cognitive impairment induced by Aβ (Quintero-Villegas et al. 2019). Aβ induces neurotoxicity through several mechanisms including apoptosis, excitotoxicity, and oxidative stress (Querfurth and Laferla 2018; Bernal-Mondragón et al. 2013). In a streptozotocin-mediated neurodegeneration murine model for AD, the intracerebroventricular (ICV) treatment of AS-19 (a 5-HT_7_R selective agonist) reduced long-term potentiation (LTP) impairment and apoptosis in hippocampal (Hashemi-Firouzi et al. 2017).

The exact mechanism of 5-HT_7_R-mediated neuroprotection in Aβ-induced neurodegeneration is currently under investigation, an effect that is likely to be mediated through multiple mechanisms.

#### Excitotoxicity

This is an important cause of neuronal damage in neurodegenerative diseases, including AD, stroke, Huntington’s disease (HD), and Parkinson’s disease (PD) (Martire et al. 2013; Lai et al. 2014; Iovino et al. 2020). Persistent excitatory -mainly glutamatergic- stimuli lead to altered calcium homeostasis, resulting in oxidative stress, mitochondrial dysfunction, disturbances in protein turnover, inflammation, and caspase-mediated apoptosis (Binvignat and Olloquequi 2020). In vitro studies suggest that the MAPK/ERK and phosphatidylinositol-3/Akt/Glycogen synthase kinase 3b pathways are closely associated with protection against glutamate-induced damage (Jiang et al. 2000; Pi et al. 2004; Li et al. 2005); modulation of these kinases via 5-HT_7_R Gαs could have therapeutic implications. 5-HT_7_R activation leads to a decrease in the NR2B and NR1 subunits of the N-methyl-D-aspartate (NMDA) glutamate receptors, thus protecting against glutamate-mediated excitotoxicity (Vasefi et al. 2013a). Treatment with LP-44, a 5-HT_7_R-specific agonist, protects human neuroblastoma SH-SY5Y cells against glutamate-mediated damage in an in-vitro model, also increasing superoxide dismutase and glutathione while decreasing TNF-α, and caspase-3 and -9 (Yuksel et al. 2019). Moreover, 5-HT_7_R modulate glutamate-NMDA activity in a time-dependent manner; while the acute activation of 5-HT_7_R by LP-12, a selective agonist of 5-HT_7_R, enhances NMDA activity, the chronic activation inhibits its activity (Vasefi et al. 2013b).

#### Oxidative stress

This can lead to membrane damage and neuronal death. (Bernal-Mondragón et al. 2013) Although, oxidative phosphorylation in mitochondria produces ROS, reactive nitrogen species, carbon-centered radicals, and sulfur-centered radicals (Pero et al. 1990), and these by-products are considered necessary for neuronal function and development (Salim 2017); increase of their levels, beyond a physiological threshold, are considered deleterious. Multiple in vitro studies have demonstrated that high levels of ROS reduce long-term potentiation, synaptic signaling, and brain plasticity (Salim 2017; O’Dell et al. 1991; Stevens and Wang 1993). Moreover, oxidative stress damages macromolecules, mainly lipid-rich structures such as membranes, via lipoperoxidation (Salim 2017).

Although no study so far has evaluated the potential effect of 5-HT_7_R in oxidative stress damage in the CNS, a study evaluating sepsis-induced lung injury demonstrated that 5-HT_7_R agonism decreased ROS burden (Cadirci et al. 2013). In contrast, the antagonism of the 5-HT_7_R by SB-269970 decreased oxidative burden in bleomycin-induced pulmonary fibrosis (Tawfik and Makary 2017). Also, 5-HT_7_R activation by LP-44 enhances microsome stability towards oxidative metabolism (Lacivita et al. 2016a). As mentioned before, 5-HT_7_R regulates the activation of ERK and Akt, and these kinases have biological effects on oxidative stress injury protection in PC12 cells (Ong et al. 2016). Thus, 5-HT_7_R may have therapeutic implications in ROS-induced neurodegeneration, something that needs to be experimentally assessed.

#### Apoptosis

Neuronal cell death is a major pathological characteristic of every neurodegenerative disease, whether via apoptosis or necrosis. In AD, both phosphorylated tau protein and Aβ aggregates induce apoptosis in *vitro* studies, with contradictory effects in tissular studies. Other proteins, including α-Synuclein (in PD or Lewy body dementia), or mutant huntingtin protein (in HD) also induce neuronal cell death via multiple mechanisms, including apoptosis (Chi et al. 2018).

It is also important to note, that apoptosis is closely related to excitotoxicity and oxidative stress; thus, these pathological effects are somewhat overlapped (Yuksel et al. 2019; Loh et al. 2006; Nicholls et al. 2007). The 5-HT_7_R agonist AS-19 reduces apoptosis in the streptozotocin-induced AD model (Hashemi-Firouzi et al. 2017). While no other study has yet evaluated this effect in the CNS, methiothepin maleate (a non-specific 5-HT_1/6/7_R agonist) prevents monocyte activation via ERK 1/2 and Nuclear factor-κB (Soga et al. 2007).

#### Long-term potentiation and long-term depression (LTD) impairment

Impairment in LTP and LTD have been extensively described in many types of dementia, such as AD (Skaper et al. 2017), PD (Marinelli et al. 2017), and HD (Filippo et al. 2007), to name a few, with a strong correlation with the cognitive symptoms in each disease. LTP and LTD are crucial for memory formation, and impairment in these are associated with amnesic and psychiatric symptoms (Loprinzi 2020).

Like other CNS effects, the role of 5-HT_7_R in LTP is controversial. Because chronically stimulated neurons by 5-HT_7_R agonists show a reduction in the expression of NMDA glutamate receptors, 5-HT_7_R has been associated with a reduction in LTP (Kobe et al. 2012b), however, 5-HT_7_R knock-out mice also display an impairment in LTP, suggesting that 5-HT_7_R receptors at a baseline state are necessary for LTP (Roberts et al. 2004). Regarding LTD, 5-HT_7_R agonism by 8-OH-DPAT (a 5-HT_1A/7_R agonist) reduced mGluR mediated LTD (Costa et al. 2012). Finally, LP-211 induces LTD on the parallel fiber-Purkinje cell synapse via Protein kinase C-MAPK pathway while impairing LTP, and pharmacological antagonism with SB-269970 decreases LTD (Lippiello et al. 2016).

#### Synaptogenesis and brain plasticity reduction

In many neurodegenerative diseases, reductions in synaptogenesis, brain plasticity, and dendritic sprouting are hallmarks of severity and progression (Querfurth and LaFerla 2010). So far, no treatment strategies have been shown to reverse that.

In vitro, activation of 5-HT_7_R in cortical and striatal forebrain neurons using LP-211 increases dendritic spine density and synaptogenesis, an effect that is abrogated by the genetical or pharmacological blockade of 5-HT_7_R (Speranza et al. 2013; Speranza et al. 2015; Speranza et al. 2017). 5-HT_7_R stimulation induces dendritic sprouting and neurite enlargement (Kvachnina et al. 2005; Rojas et al. 2014; Canese et al. 2015), an effect probably mediated by effectors of both, Gas and Ga12 signaling pathways (Volpicelli et al. 2014).

#### Neurotrophin depletion

Neurotrophins like BDNF and platelet-derived growth factor (PDGF)-β are necessary for the development and correct function of the CNS. Depletion or altered signaling occurs in neurodegenerative diseases (Kashyap et al. 2018) AD, PD, HD, and FTD are associated with a reduction in the expression of BDNF and other neurotrophins, where the modulation of BDNF could have a potential therapeutic role (Palasz et al. 2020; Schulte-Herbrüggen et al. 2008; Alberch et al. 2002; Huey et al. 2020). Interestingly, 5-HT_7_R modulates both neurotrophins and their receptors. 5-HT_7_R activation by LP-12 leads to an increase in PDGF-β, with increased protection against glutamate-mediated excitotoxicity (Vasefi et al. 2013a). In addition, 5-HT_7_R agonism by LP-12 increases the expression and affinity of the tropomyosin-related kinase B receptor, one of the receptors for BDNF (Samarajeewa et al. 2014).

#### Immune-mediated damage

The specific role of 5-HT_7_R as neuro-immune mediators is still debated. 5-HT_7_R is expressed broadly by different immune cells, including monocytes, lymphocytes, and dendritic cells, but its anti-inflammatory potential has been shown in some but not all studies (Quintero-Villegas and Valdés-Ferrer 2019).

### Potential disease-specific role of 5-HT_7_R in neuropsychiatric illness

#### Neuronal hyperexcitability and seizures

Epilepsy-induced neuronal damage shares pathophysiological neurodegenerative features with dementias and other CNS diseases, such as increased inflammation and excitotoxicity (Nikiforuk 2015). Epilepsy is prevalent in sufferers of CNS diseases, including AD, PD, or HD, and, when present, indicates a higher burden of neurodegeneration (Cano et al. 2021).

5-HT_7_R manipulation has shown controversial results in pre-clinical studies of epilepsy. Non-specific pharmacological blockade of 5-HT_7_R reduces the prevalence of audiogenic seizures in a DBA/2J rat model (Bourson et al. 1997).

Additionally, SB-258719, an antagonist of 5-HT_7_R showed a benefit in reducing spontaneous epileptic activity in a WAG/Rij rat model of absence seizures (Graf et al. 2004). Finally, in a pilocarpine-induced rat model of temporal lobe epilepsy, AS-19 increased epileptic activity, whereas SB-269970, an antagonist of 5-HT_7_R reduced seizure activity. Interestingly, the expression of 5-HT_7_R was higher in the epilepsy group, compared to the control group (Yang et al. 2012). However, in a pilocarpine-induced model of epilepsy, the density of 5-HT_7_R decreased in the hippocampus, especially in the dentate gyrus (Núñez-Ochoa et al. 2021). Hence, the role of 5-HT_7_R in epilepsy is still unclear but avidly explored. The controversial finding may represent that 5-HT_7_R plays different roles in different models of epilepsy. Evaluation of 5-HT_7_R tissue expression in specimens obtained from patients undergoing surgical excision of epileptic foci may help to clarify this controversy.

#### Mood disorders

The relationship between mood disorders and cognitive disorders is a topic of continuous research. The prevalence of depression and anxiety is higher among patients with dementia, and patients with depression have a higher prevalence of dementia (Lyketsos et al. 2002). Of therapeutic relevance, even mild levels of depression can impact substantially the functionality and quality of life of patients with dementia. Thus, treating these symptoms may be crucial in the management of dementia (Gutzmann and Qazi 2015).

In a rat model of stress using forced swim and tail suspension, pharmacological and genetic blockade of 5-HT_7_R reduced depressive symptoms and improved REM sleep (Hedlund et al. 2005). 5-HT_7_R KO mice show improved mobility in the Porsolt swim test; however, the pharmacological blockade by SB-258719 only had the same results when rats were tested in the dark (Guscott et al. 2005). Similar results were observed with SB-269970 in depression and anxiety, with an effect similar to the one observed with imipramine (Wesołowska et al. 2006). In experimental depression, pharmacological blockade of 5-HT_7_R seems to have a rapid effect in reducing depressive symptoms (Mnie-Filali et al. 2011). Altogether, while more data is needed before moving to the clinical trial setting, these experimental models suggest that 5-HT_7_R may be a druggable target for depression.

The calcium-binding protein S100B interacts with 5-HT_7_R and negatively regulates inducible cAMP accumulation; its overexpression in transgenic mice is associated with depressive-like symptoms, which are reversed by the administration of SB269970 (Stroth and Svenningsson 2015).

The non-specific blockade of 5-HT_7_R with HBK-15 (5-HT_7_R, 5-HT_1A_R, and 5-HT_3_R antagonist) (Pytka et al. 2018), aryl sulfonamide derivatives of dihydro benzofuran oxy)ethyl piperidines (a2 and 5-HT_7_R antagonist) (Canale et al. 2021), and lurasidone (5-HT_2A_R and 5-HT_7_R antagonist) (Woo et al. 2013), to name a few, have similar effects regarding depression and anxiety.

#### The contradictory memory effect of 5-HT_7_R

Evidence about the effect of 5-HT_7_R on learning and memory is still contradictory. Systemic administration of LP-211 and AS-19 revert scopolamine-induced amnesia and enhanced auto-shaping training learning respectively in rats; whereas pharmacologic blockade with SB-269970 has the opposite effect (Perez-García and Meneses 2005). Accordingly, LP-211 administrated intraperitoneally improves long-term memory, with conditioned responses up to 80%, compared to 20–30% in the control groups, with no effect on short-term memory, and reverts scopolamine-induced memory impairment, something associated with increased cAMP levels (Meneses et al. 2015). Moreover, 5-HT_7_R agonism appears to revert the memory impairment mediated by 5-HT_1A_R, based on the fact that the co-administration of SB-269970 and 8-OH-DPAT (a 5-HT_7_R/5-HT_1A_R agonist) caused greater performance impairment in contextual learning than the administration of 8-OH-DPAT alone (Eriksson et al. 2008).

On the other hand, SB-269970 improved reference memory in a radial arm maze task (Gasbarri et al. 2008). In agreement, the administration of SB-656104-A (a 5-HT_7_R-specific antagonist) reverses dizocilpine-induced memory impairment induced, with significant differences in thigmotaxic swimming in the Morris water maze test (Horisawa et al. 2011). We have observed that intracerebroventricular injection of LP-211 reverts memory impairment induced by Aβ without a discernible effect on healthy rats (Quintero-Villegas et al. 2019). The answer to this paradox is inconclusive with the current evidence (Meneses 2014; Stiedl et al. 2015; Zareifopoulos and Papatheodoropoulos 2016), suggesting a memory type-specific role of 5-HT_7_R.

### Lack of clinical trial-derived data

No clinical trials with 5-HT_7_R-specific modulation for neurodegenerative studies have been performed. However, the potential effect of non-specific serotonin activation in neurodegenerative diseases is a highly studied topic and the outflow of human data may start shortly. Interestingly, lurasidone, a 5-HT_7_R antagonist, with an effect on other serotonin receptors and D2 dopamine receptor (Meltzer et al. 2020), has been FDA approved for the treatment of schizophrenia (Okubo et al. 2021).

## Conclusion

At this point, no 5-HT_7_R-specific drugs have been evaluated for neurodegenerative diseases, and few studies have experimentally evaluated their potential therapeutic effects. Hence, elucidating the effect of 5-HT_7_R in health and (predominantly neurodegenerative) diseases may have vast translational and therapeutic implications. 5-HT_7_R agonists may be neuroprotective by acting at multiple levels, including the reduction of excitotoxicity and oxidative stress, synaptic remodeling, regulation of neurotrophic factors, or immunomodulation, to name a few. The need for more studies, both experimental and clinical, before reaching conclusions about a therapeutic role for this serotonin receptor cannot be overemphasized.

## Data Availability

All data were extracted from the PubMed database using the MeSH established in the “Methods” section.

## Abbreviations

5-HT: 5-Hydroxytryptamine/serotonin
5-HT1AR: 5-HT1A receptor
5-HT7R: 5-HT7 receptor
AD: Alzheimer’s disease
Aβ: Amyloid β
BDNF: Brain-derived neurotrophic factor
cAMP: Cyclic adenosine monophosphate
Cdc42: Cell division control protein 42
CNS: Central nervous system
ERK: Extracellular signal-regulated kinase
FTD: Frontotemporal dementia
GPCR: G-protein-coupled receptor
HD: Huntington’s disease
ICV: Intracerebroventricular
IL: Interleukin
LTD: Long-term depression
LTP: Long-term potentiation
MAPK: Mitogen-activated protein kinase
PD: Parkinson’s disease
PDGF: Platelet-derived growth factor
ROS: Reactive oxygen species
TNF: Tumor necrosis factor
Trkb: Tropomyosin-related kinase B
